# Miller Fisher Syndrome With Positive Anti-GQ1b/GQ1d Antibodies Associated With COVID-19 Infection: A Case Report

**DOI:** 10.7759/cureus.36924

**Published:** 2023-03-30

**Authors:** Mohammad Abu-Abaa, Omar Jumaah, Aliaa Mousa, Alaa Aldookhi

**Affiliations:** 1 Internal Medicine, Capital Health Regional Medical Center, Trenton, USA

**Keywords:** dysarthria, dysphagia, covid-19, miller-fisher syndrome, gullian-barre syndrome

## Abstract

The association between Guillain-Barré Syndrome (GBS) and its variants including Miller Fisher syndrome (MFS) has been reported and debated in the literature. Herein, we are reporting a 59-year-old male patient who had flu-like symptoms for 10 days prior to presentation with rapidly progressive weakness, dysphagia, and dysarthria. He tested positive for COVID-19 and further workup showed positive anti-GQ1b and GQ1d antibodies. The diagnosis of MFS was presumed and prompted the commencement of intravenous immunoglobulin (IVIG). Respiratory deterioration prompted intubation and failure of extubation necessitated plasmapheresis. This treatment culminated in successful extubation and discharge to a long-term care facility. This case adds to the currently limited body of cases that report the association of a rare GBS variant with COVID-19 infection. Only a few of the reported cases of COVID-19-related MFS cases had positive anti-GQ1b antibodies. This may well be the first reported case of COVID-19-related MFS with positive anti-GQ1b and anti-GQ1d antibodies.

## Introduction

Miller Fisher syndrome (MFS) is a rare form of the more commonly encountered acute inflammatory demyelinating polyneuropathy (AIDP) or Guillain-Barre syndrome (GBS), an autoimmune disease of the peripheral nerves. Unlike GBS with a typical ascending paralysis, MFS is characterized by descending paralysis with a classical triad of ophthalmoplegia, ataxia, and areflexia. Ophthalmoplegia can be explained by the involvement of the third, fourth, and sixth cranial nerves, while ataxia can be explained by cerebellar involvement. Areflexia can be explained by lower motor neuron involvement [1]. The association between MFS and COVID-19 infection has been reported in the literature [2,3]. The severity of COVID-19 symptoms has ranged from asymptomatic to severe (requiring hospitalization) [2,3].

## Case presentation

A 59-year-old male patient presented to the emergency department (ED) with a one-day history of weakness. Initially, he noticed weakness of the lower extremities that progressed throughout the same day to affect all limbs as well as associated generalized tingling with slurred speech, unsteady gait, and dysphagia. Around 24 hours later, he was not able to sit up from a supine position. His past medical history was unremarkable but a history of flu-like symptoms affecting multiple members of his household was reported, starting one week prior to presentation. Although the conditions of his household members had improved, he was still complaining of dry cough, anosmia, fever, chills, and shortness of breath. He denied diarrhea, abdominal pain, nausea, and vomiting. He received three doses of the COVID-19 vaccine. He denied illicit drug use or receiving recent vaccines. He also denied recent travel, surgery, and trauma. The only medication in use was acetaminophen. In ED, vital signs included a temperature of 36.8 degrees Celsius, heart rate of 72 beats/minute, respiratory rate of 18 cycles per minute, blood pressure of 155/95 mmHg, and SpO2 of 96% on room air. On physical examination, he was fully oriented, had bilateral facial droop and ptosis, dysarthria, nondilated reactive pupils bilaterally, bilateral ocular movement impairment in all directions, intact visual fields bilaterally, mild weakness on palatal elevation, dysmetria on finger nose-test bilaterally, 3/5 MRC muscle power on all four extremities, normal tone, generalized areflexia as well as diminished light touch, pinprick, and temperature sensations on all extremities, and impaired heel-shin test bilaterally. 

**Figure 1:**
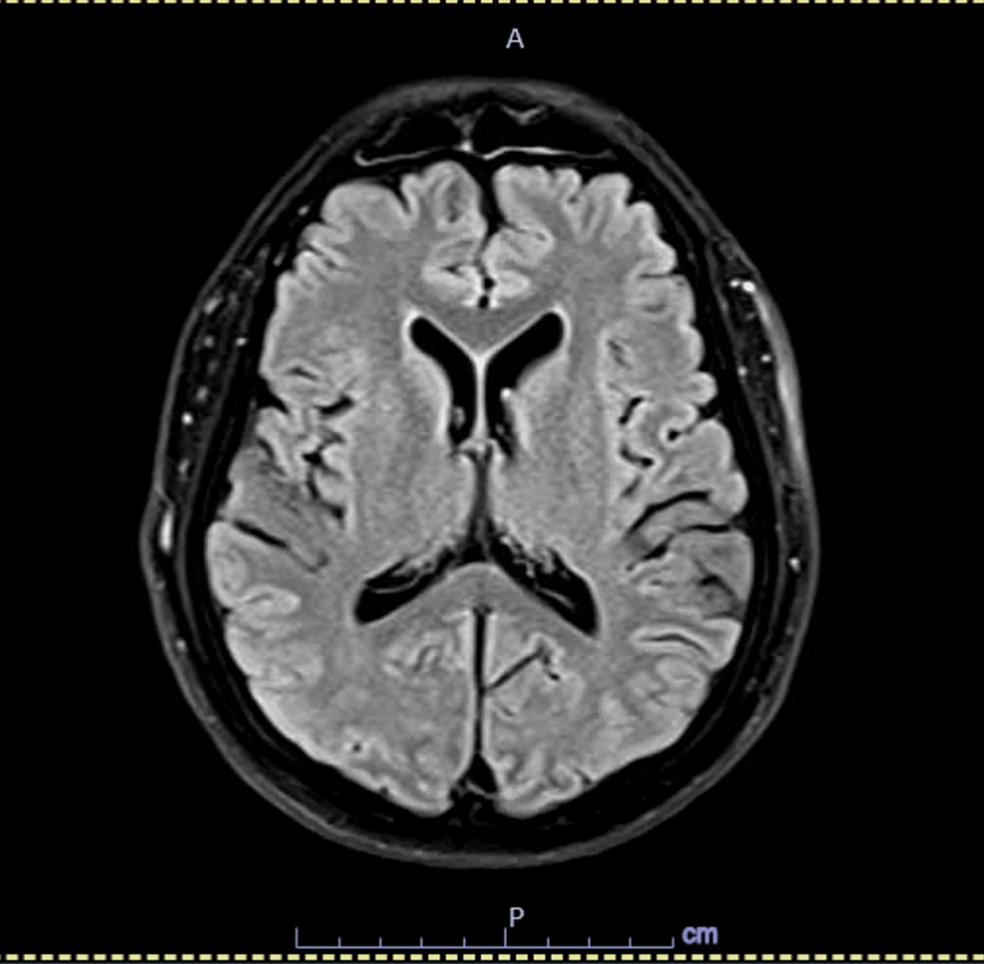
MRI Brain Magnetic resonance imaging (MRI) fluid attenuation inversion recovery (FLAIR) sequence of the brain with no reported obvious pathology.

**Figure 2:**
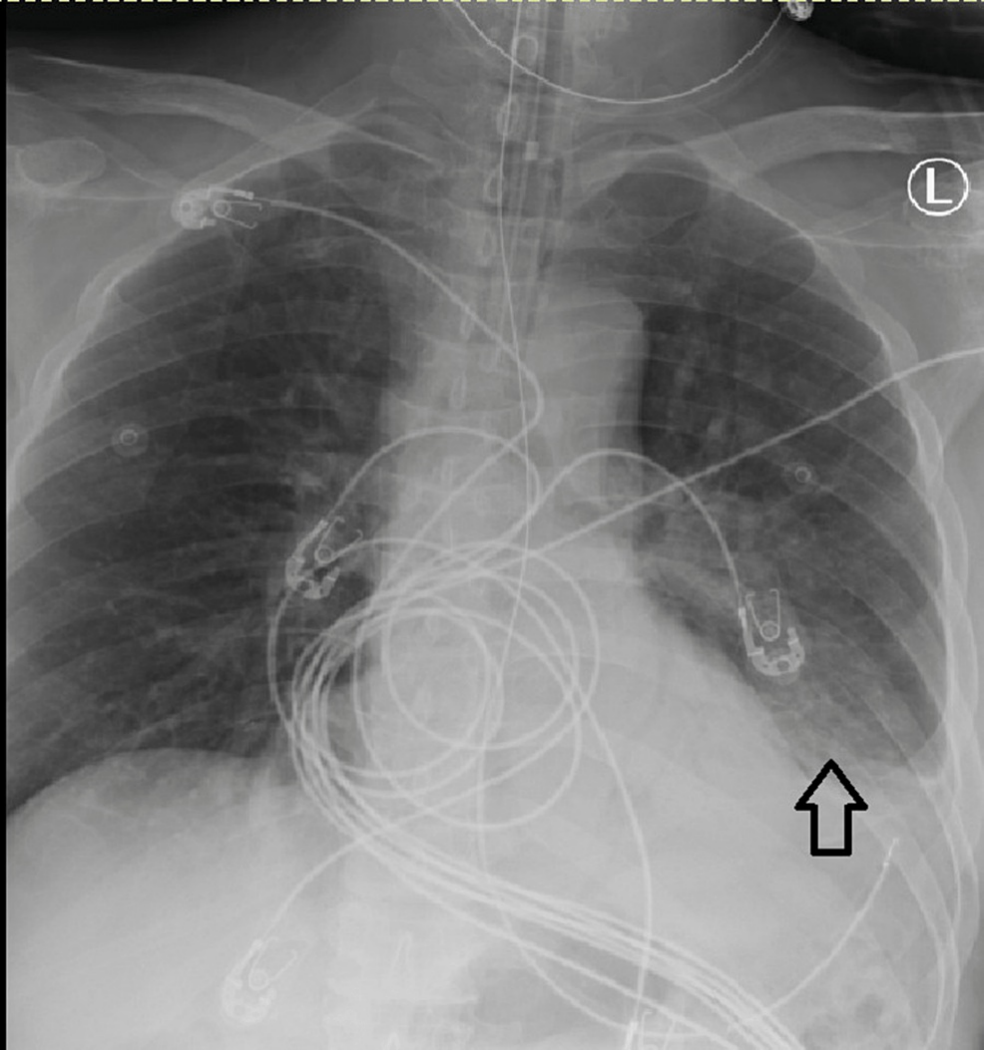
Chest X-ray A posterior-anterior chest X-ray showing a left-sided basal infiltrate (arrow).

**Figure 3:**
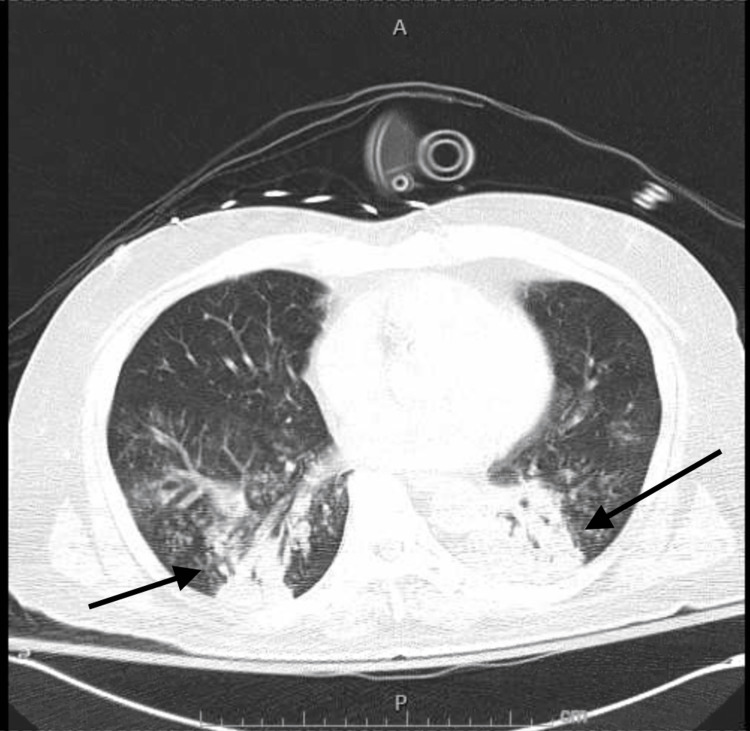
Computed tomography chest Computed tomography (CT) scan of the chest showing bilateral basal infiltrate (arrows).

Basic labs were unremarkable. A stroke work-up including CT scans of the head, CT brain perfusion, and CT angiography of the brain and neck were all unremarkable. Magnetic resonance imaging (MRI) brain with/without contrast was unremarkable (Figure 1). MRI of the cervical spine with/without contrast was also unremarkable. COVID-19 PCR was reactive and chest X-ray was remarkable for left basilar infiltrate (Figure 2). Negative inspiratory force (NIF) was 38 on presentation. CT chest was also obtained and showed bilateral basal infiltrates (Figure 3). An extensive respiratory panel was negative. The patient was started empirically on intravenous immunoglobulin (IVIG) for high suspicion of GBS/AIDP.

He was outside the window for remdesivir consideration. Corticosteroids were also not pursued due to the risk of progression of GBS/AIDP to chronic inflammatory demyelinating polyneuropathy (CIDP). Less than 24 hours later, his respiratory function deteriorated with progressive hypoxia and hypercapnia, prompting intubation. Lumbar puncture (LP) yielded 19 ml of cerebrospinal fluid with an opening pressure of 19 cm H2O. Cerebrospinal fluid (CSF) analysis showed clear CSF with white blood cells (WBC) of 9 cells/ml, normal protein at 26 mg/dl, and elevated glucose at 95 mg/dl. CSF culture was negative. Extensive infectious analysis of the CSF was negative.

Antiacetylcholine receptor as well as anti-MUSK antibodies were negative. A paraneoplastic panel including anti-Hu, anti-Yo, and anti-Ri was negative. Anti-LGI1, anti-CASPR2, anti-AGNA-1, anti-CRMP-5, anti-amphiphysin, anti-Purkinje cell type 2, and Tr antibodies were also negative. Blood, urine, and sputum cultures remained negative. Slow improvement of muscle weakness was noticed after five days of IVIG which was continued for two more days. Anti-GQ1b and anti-GQ1d antibodies were reactive. Failure of extubation with residual diaphragmatic weakness prompted the administration of five sessions of plasmapheresis. His muscle improvement was slow and his muscle power was 2/5 on all extremities. Extubation was feasible to tracheostomy and the patient was discharged to a long-term care facility.

## Discussion

Neurological manifestations of COVID-19 infection are reported in up to 36.4% of COVID-19 cases [4]. They can vary widely and range from non-specific symptoms such as headache and dizziness to more serious complications including ischemic and hemorrhagic stroke, myelitis, and cerebral venous sinus thrombosis [5]. COVID-19 also has been linked to both viral and autoimmune encephalitis [5]. Encephalopathy has been reported in 20-30% of COVID-19 patients at the time of admission or during hospitalization. This increases to 60-70% in critically ill patients [6]. Neuroradiological findings typical of posterior reversible encephalopathy syndrome (PRES) have been reported in more than 1% of COVID-19 patients and are usually attributed to renal dysfunction and/or hypertension [7]. The prevalence of seizures is 0.5-1.4% [8]. GBS is the most common neuromuscular complication of COVID-19, which has been observed in both parainfectious and postinfectious patterns [9]. GBS/MFS have been reported as the initial presentation of COVID-19 infection [10,11]. 

GBS has an incidence rate of one to two cases per million adults and 0.4-1.4 per 100,000 children [12]. MFS has been reported rarely in association with COVID-19 infection [2] and accounts only for 5% of GBS cases [13]. The most common trigger of GBS/MFS is the preceding viral infection, most commonly of the respiratory or gastrointestinal tract [13]. The most common variant of GBS in COVID-19 patients is acute inflammatory demyelinating polyneuropathy (AIDP) in 75% of cases, followed by acute motor axonal neuropathy in 11% and acute motor-sensory axonal polyneuropathy in 7% of cases [14]. GBS/MFS have been reported also after COVID-19 vaccines [11,15]. 

The diagnosis of MFS is usually a clinical one. There is no difference in clinical presentation between COVID-19-related cases and classical ones. Positive serology including anti-GQ1b antibody is supportive. It is an autoantibody directed against ganglioside GQ1b, which is abundant at paranodal regions at nodes of Ranvier along myelinated axons including cranial nerves [13]. This has a sensitivity of 85% [16]. However, the majority of COVID-19-related cases of MFS have a negative serology according to a recent systematic review [17]. On the other hand, the occurrence of anti-GQ1d antibodies has been only rarely reported in association with MFS/GBS. This has been reported less often in MFS as compared to other GBS variants, although it has been reported in cases of MFS with negative anti-GQ1b antibodies [18]. As in the case of our patient, most of the reported cases of COVID-19-related MFS have no findings on MRI [2]. 

As the angiotensin-converting enzyme (ACE) receptors are widely spread throughout the nervous system, it is pathologically plausible to connect COVID-19 infection and neurological complications including GBS/MFS through direct neurological invasion and/or immune-mediated neurological damage [17]. The longest reported latency period between the onset of COVID-19 symptoms and the onset of GBS/MFS is 10 weeks [19]. A recent systematic review of seven cases of COVID-19-associated MFS showed a median latency period of 14.75 days [18]. This was confirmed by another systematic review that showed anti-GQ1b at 71.4% and anti-GD1b at 20% [18]. In addition to our case, there are only four reported cases of COVID-19-related MFS with positive anti-GQ1b antibodies [11]. In immune-mediated cases of MFS, anti-GQ1b antibody is believed to play a role in symptoms pathogenesis through molecular mimicry as it was found to bind to certain infectious triggers of GBS/MFS such as *Campylobacter jejuni and Haemophilus influenzae* [20,5]. This raises the possibility of a similar mechanism in COVID-19-related GBS/MFS. Parainfectious patterns may be explained by initial subclinical manifestations of COVID-19. 

Although electromyography (EMG) and nerve conduction studies were not carried out on our patient, the clinical presentation and positive serology are highly suggestive of MFS. Cerebrospinal fluid (CSF) analysis was likely a false negative as it was obtained early in the course of the disease. Similar to other reports of COVID-19-related GBS/MFS, the relationship between COVID-19 infection and MFS, in this case, is based on a temporal relationship and lack of other etiologies. It is also important to mention that a retrospective population-based cohort study in the UK investigating the relationship between COVID-19 and GBS has failed to show a causal relationship [21]. Further studies are needed to ascertain this conclusion. 

There is no difference in treatment between COVID-19-related cases of GBS/MFS and other cases. A recent systematic review showed that 78% of cases had a good response to IVIG [22]. This indicates a similar response to IVIG among those with COVID-19-related GBS/MFS to classical GBS/MFS. To the best of our knowledge, 11 cases of COVID-19-related MFS were reported in the literature. Table 1 has a summary of these cases.

**Table 1:** A summary of reported COVID-19-related Miller Fisher syndrome cases

Citation	Age	Gender	COVID-19 Severity	Latency Period	Serology	Treatment	Outcome
Kuang et al. [19]	26	Male	Mild	10 weeks	Not reported	IVIG	Near complete recovery
Yaqoob et al. [23]	22	Male	Mild	3 days	Not reported	IVIG	Complete recovery
Ray et al. [15]	63	Male	Mild	1 Day	Not reported	IVIG	Not reported
Zayet et al. [24]	25	Female	Asymptomatic	MFS on Presentation	Negative anti-GQ1b	IV Thiamine	Near complete recovery
Fernández-Domínguez et al. [25]	74	Female	Hospitalized	12-15 Days	Negative anti-GQ1b	IVIG	Near complete recovery
Gutiérrez-Ortiz et al. [26]	50	Male	Mild	5 Days	Positive anti-GQ1b	IVIG	Near complete recovery
Gutiérrez-Ortiz et al. [26]	39	Male	Mild	3 Days	Not reported	Acetaminophen	Complete recovery
Lantos et al. [27]	36	Male	Mild	4 Days	Equivocal anti-GM1 and negative anti-GQ1b	IVIG	Not reported
Manganotti et al. [3]	50	Female	Hospitalized	16 Days	Negative all antiganglioside antibodies	IVIG	Near complete recovery
Reyes-Bueno et al. [28]	51	Female	Mild	15 Days	Not reported	IVIG	Not reported
Senel et al. [29]	61	Male	Mild	27 Days	Negative anti-GQ1b	IVIG	Complete

## Conclusions

GBS/MFS should always be considered in those with unexplained neurological deficits in the setting of COVID-19 infection. This is possibly the first reported case of COVID-19-related MFS with positive anti-GQ1b and antI-GQ1d antibodies, indicating the potential molecular mimicry and cross-reactivity between these immunological targets and COVID-19. This may provide insight and possibly explain the connection between these entities. 
